# Changes in Risk of Immediate Adverse Reactions to Iodinated Contrast Media by Repeated Administrations in Patients with Hepatocellular Carcinoma

**DOI:** 10.1371/journal.pone.0076018

**Published:** 2013-10-02

**Authors:** Naoto Fujiwara, Ryosuke Tateishi, Masaaki Akahane, Masataka Taguri, Tatsuya Minami, Shintaro Mikami, Masaya Sato, Kouji Uchino, Kenichiro Enooku, Yuji Kondo, Yoshinari Asaoka, Noriyo Yamashiki, Tadashi Goto, Shuichiro Shiina, Haruhiko Yoshida, Kuni Ohtomo, Kazuhiko Koike

**Affiliations:** 1 Department of Gastroenterology, Graduate School of Medicine, The University of Tokyo, Bunkyo-ku, Tokyo, Japan; 2 Department of Radiology, Graduate School of Medicine, The University of Tokyo, Bunkyo-ku, Tokyo, Japan; 3 Department of Biostatistics and Epidemiology, Graduate School of Medicine, Yokohama City University, Yokohama, Kanagawa, Japan; Icahn School of Medicine at Mount Sinai, United States of America

## Abstract

**Background:**

To elucidate whether repeated exposures to iodinated contrast media increase the risk of adverse reaction.

**Materials and Methods:**

We retrospectively reviewed 1,861 patients with hepatocellular carcinoma who visited authors’ institution, a tertiary referral center, between 2004 and 2008. We analyzed cumulative probability of adverse reactions and risk factors. We categorized all symptoms into hypersensitivity reactions, physiologic reactions, and other reactions, according to the American College of Radiology guidelines, and evaluated each category as an event. We estimated the association between hazard for adverse reactions and the number of cumulative exposures to contrast media. We also evaluated subsequent contrast media injections and adverse reactions.

**Results:**

There were 23,684 contrast media injections in 1,729 patients. One hundred and thirty-two patients were excluded because they were given no contrast media during the study period. Adverse reactions occurred in 196 (0.83%) patients. The cumulative incidence at 10^th^, 20^th^, and 30^th^ examination was 7.9%, 15.2%, and 24.1%, respectively. Presence of renal impairment was found to be one of risk factors for adverse reactions. The estimated hazard of overall adverse reaction gradually decreased until around 10^th^ exposure and rose with subsequent exposures. The estimated hazard of hypersensitivity showed V-shaped change with cumulative number of exposures. The estimated hazard of physiologic reaction had a tendency toward decreasing and that of other reaction had a tendency toward increasing. Second adverse reaction was more severe than the initial in only one among 130 patients receiving subsequent injections.

**Conclusion:**

Repeated exposures to iodinated contrast media increase the risk of adverse reaction.

## Introduction

Hepatocellular carcinoma (HCC) is the sixth most prevalent cancer and the third most frequent cause of cancer-related death [1] and the incidence of HCC is increasing over the last decade [2,3]. Patients with HCC receive repeated contrast media (CM) injections, not only for diagnosis but also for surveillance of recurrence after initial complete treatment by surgery or local ablation [4]. This is because the residual liver tissue is usually already damaged by chronic liver disease and intrahepatic recurrence is very frequent [5].

The non-ionic CM have lower osmolality and tend to have fewer side effects, while retaining satisfactory radiographic opacification [6,7], and are thus have already completely replaced the older ionic higher osmolality contrast media for intravascular use. Previous studies have reported the rate of adverse reactions (ARs) to non-ionic CM to be from 1 to 4% [8–10]. The severity ranges from mild symptoms, such as urticaria and pruritus, to more severe reactions, such as cardiopulmonary arrest and even death [11]. However, because mild ARs are generally tolerated, and moderate-to-severe reactions are very rare, as many as 60 million doses of iodinated CM are used worldwide each year [12].

Although the etiology of many or most hypersensitivity reactions is unknown, an allergic-like mechanism appears to be engaged in some patients because allergic individuals or patients with asthma are considered to be at an increased risk for developing AR [13]. Therefore, it is possible that repeated exposures to iodinated CM increase the risk of AR by a mechanism based on sensitization. Especially, HCC patients, who receive multiple injections to CM, are likely to be put at the risk of ARs. However, to our knowledge, no reports have investigated whether the risk of ARs increases with the cumulative number of examinations with iodinated CM in individual patients with HCC.

The aim of this study was to evaluate the risk factors for AR to non-ionic iodinated CM, among a large number of contrast-enhanced computed tomography (CT) examinations and, and to elucidate whether repeated exposures to CM increase the risk of AR upon subsequent exposure in patients with HCC.

## Materials and Methods

### Ethic statement

This retrospective study was conducted according to the ethical guidelines for epidemiological research designed by the Japanese Ministry of Education, Culture, Sports, Science and Technology and Ministry of Health, Labour, and Welfare. The study design was included in a comprehensive protocol of retrospective study at the Department of Gastroenterology, The University of Tokyo Hospital approved by The University of Tokyo Medical Research Center Ethics Committee (approval number 2058). The following statements were posted at a website (http://gastro.m.u-tokyo.ac.jp/med/0602A.htm) and participants who do not agree to the use of their clinical data can claim deletion of them.

Department of Gastroenterology at The University of Tokyo Hospital contains data from our daily practice for the assessment of short-term (treatment success, immediate adverse events etc.) and long-term (late complications, recurrence etc.) outcomes. Obtained data were stored in an encrypted hard disk separated from outside of the hospital. When reporting analyzed data, we protect the anonymity of participants for the sake of privacy protection. If you do not wish the utilization of your data for the clinical study or have any question on the research content, please do not hesitate to make contact with us.

### Patients

The medical records of 1,861 consecutive patients with HCC who visited the authors’ institution between January 1, 2004 and December 31, 2008 were reviewed retrospectively. Most of these patients received contrast-enhanced CT examinations on a regular basis for the assessment of disease status, including HCC recurrence. We counted the number of cumulative contrast-enhanced CT examinations and transarterial chemoembolization (TACE) treatments with intra-arterial infusion of iodinated CM, until death, drop-out, or December 31, 2011, whichever came first.

We collected the following data recorded on the application form for contrast-enhanced CT or TACE: sex, age, past history of ARs to iodinated CM, past history of asthma, history of other allergies, corticosteroid use and the presence of renal impairment, defined as an estimated glomerular filtration rate (eGFR) < 60 ml min−1, as an indicator of chronic renal insufficiency [14]. We also evaluated hepatic function based on the Child-Pugh classification (CPC) [15] to assess its association with the incidence of AR.

### Contraindications for contrast media administrations and prophylaxis

Pregnant women, patients with previous severe AR to CM and patient with severe thyroid disease are contraindicated to administration of CM at our institution. Patients with asthma, renal impairment, severe heart disease, macroglobulinemia, multiple myeloma and pheochromocytoma are also in principle excluded from an indication for CM administration. However, in clinical practice, patients with these impairments or diseases sometimes receive CM when the clinical advantages outweigh the potential toxicity.

Corticosteroid is sometimes given prophylactically to patients with previous mild AR to CM and patients with mild asthma when they are exposed to iodinated CM. In this study, the decision whether or not to utilize corticosteroid prophylaxis was made by individual clinicians, based on the available information [16–18].

### Contrast-enhanced CT and TACE

All contrast-enhanced CT examinations and TACE, performed on HCC patients at the authors’ hospital from January 1, 2004 through December 31, 2011, were identified by means of an electronic query to the institutional database system. The following non-ionic low osmolality CM had been in use: iopamidol 300-mg iodine per mL (Iopamiron 300; Nihon Schering, Osaka, Japan), iopamidol 370-mg iodine per mL (Iopamiron 370; Nihon Schering, Osaka, Japan), or iohexol 350-mg iodine per mL (Omnipaque 350; Daiichi Sankyo, Tokyo, Japan). The choice of iodinated CM depended on the protocol and the attending physician. When a patient with a previous immediate AR to iopamidol required further examination, we switched to iohexol and vice versa in accordance with the guidelines set forth by the European Society of Urogenital Radiology [19].

CT scanning was performed according to standard clinical protocols. The standard volume of 2 mL kg−1, to a maximum of 100 mL, was injected at a rate of 2.3–3.3 mL s−1 with a power injector. All patients were monitored closely during injection. If any symptom was observed during or immediately after CM injection, the clinician or radiologist checked the patient’s vital signs and performed a physical examination.

TACE was performed under local anesthesia. A catheter was inserted into the hepatic artery via the femoral artery. When hypervascular tumors were identified during the hepatic angiography procedure, the feeding arteries were selectively embolized with gelatin sponge particles after an emulsion of epirubicin hydrochloride (Pharmorubicin; Pfizer Japan, Tokyo, Japan), and iodized oil (Lipiodol Ultra-Fluid; Schering Japan, Osaka, Japan) was injected under X-ray monitoring.

### Contrast-enhanced MRI and contrast-enhanced ultrasonography at our institution

Contrast-enhanced magnetic resonance imaging (MRI) and contrast-enhanced ultrasonography (CEUS) are efficient tools for detecting HCC [20,21]. However, MRI requires more time than CT, and there are fewer MRI devices than CTs in our institution. Thus, we were compelled to limit the number of patients examined by MRI. Along with B-mode ultrasonography, a comprehensive assessment of the whole liver by CEUS may sometimes be hampered by the patient’s body habitus, colonic interposition, or morphologic changes by cirrhosis, which leads to decreasing the ability of detecting small HCCs [22]. Accordingly, contrast-enhanced CT is the first choice for diagnosis of HCC and follow-up after the therapy at our institution unless the patient has contraindication for iodinated CM administration. We sequentially use contrast-enhanced MRI or CEUS for the difficult cases to distinguish HCC from benign nodules [23] or to detect HCC by only dynamic CT [24].

### Adverse reactions to contrast media

This study was focused on ARs related to iodinated CM. However, it is difficult to discriminate between ARs to CM and accidental events. Therefore, after considerable preliminary evaluation of the data, we concluded that the following view of ARs was useful and statistically sound. First, we evaluated any symptom as an AR, regardless of whether the symptom was attributable to CM, to the procedure, or an accidental event. Second, we categorized all symptoms into hypersensitivity reactions, physiologic reactions, and ‛other’ reactions, according to the American College of Radiology (ACR) guidelines, and evaluated each category as an event [25]. Hypersensitivity reactions included skin symptoms such as pruritus and urticaria, angioedema (such as a scratchy throat, slight throat and/or facial swelling), bronchospasm, and anaphylactoid shock, that is, hypotension with tachycardia. ACR guidelines categorized paroxysmal sneezing as a hypersensitivity reaction. However, due to the difficulty in distinguishing between allergic sneezing and coincidence, we categorized all sneezing into other reactions. Other reactions, not specifically outlined in the ACR guidelines, but that were sometimes observed in this study, were defined as symptoms that were observed immediately after CM injection, although it was unclear whether the symptoms were related to CM injections. Specifically, this category included sneezing and cough. The severity of ARs to iodinated CM was classified according to ACR guidelines.

The radiologists and physicians attending the CT examination and TACE reported on the manifestation and severity of the AR, and whether the patient received medical treatment. These data were fed immediately into the electronic medical record system shared by hospital staff. If more than one manifestation of the AR to a single CM injection was identified, we chose the most severe reaction as the manifestation.

### Subsequent contrast media injections and adverse reactions to them

Some patients who exhibited an AR subsequently received another CM injection and experienced subsequent ARs. Thus, we investigated the association between the initial ARs and the reactions to subsequent injections. We also elucidated manifestations and the severity of subsequent reactions and, if present, incremented its severity.

### Statistical method

We retrospectively collected the demographic and laboratory data that were available 2 weeks before the initial CM examination. The difference was assessed using the Mann–Whitney U-test for continuous data; the chi-squared test or Fisher’s exact test was used for categorical data. The cumulative probability curves were constructed using the method of Kaplan–Meier, which were compared with the log-rank test. We also analyzed the cumulative incidence of ARs with respect to each AR category described above (i.e., allergic, physiologic, or other) and compared this using the log-rank test. We did not treat these categories as competing risks, because these reactions may have occurred at a single administration.

To investigate whether the risk of AR increased with accumulated exposure to CM, we analyzed the relationship between the hazard function for AR to CM and the number of cumulative exposures to CM. The hazard function, defined as h(t) = −S′(t)/S(t), where S(t) is the survival function, and S′(t) its derivative, is a measure of the tendency for the event to occur. By calculating the hazard function for the occurrence of AR, with respect to the number of previous exposures as a function of time, we can evaluate whether or not the number of previous exposures affected the immediate risk of AR. In this analysis, the cumulative number of exposures to CM had a major influence on the results. Thus, we calculated the hazard functions for only the ‛exposure-naïve’ patients, defined as those who had their initial injection of iodinated CM during the study period. Additionally, we used restricted quadratic splines in the Poisson-rate regression model, with three knots at the 10th, 50th, and 90th percentiles of the number of cumulative exposures to CM, to investigate the possible nonlinear relationship between the number of cumulative exposures to CM and the hazard function [26,27].

Risk factors for AR were analyzed by univariate and multivariate Cox proportional hazard-regression models. A stepwise variable selection was performed with Akaike Information Criteria in a multivariate analysis. The results of the multivariate analyses were presented as hazard ratios (HR), with their corresponding 95% confidence intervals (CI) and P values from the Wald test.

In clinical practice, we often do not know the cumulative number of iodinated CM injections a patient has been administered; but instead, are aware of the presence or absence of past injection. Hence, we included past exposure to iodinated CM before the study period as a covariate, in addition to the following variables: age (as a continuous number), sex, presence of a previous AR to iodinated CM, presence of allergy, presence of asthma, presence of renal impairment, defined as an estimated glomerular filtration rate (eGFR) < 60 ml min−1, as an indicator of chronic renal insufficiency [28], presence of corticosteroid use, and CPC A vs. B or C.

Statistical analyses were performed using R 2.13.0 (http://www.R-project.org) and SAS (Cary, NC). All tests were two-sided, with P < 0.05 denoting statistical significance.

## Results

Overall, 1,861 patients with HCC were reviewed, of which 132 were excluded because they were given no CM during the study period. The baseline characteristics of the remaining 1,729 (92.9%) patients are summarized in Table 1. Nine hundred and seventy-three (56.3%) patients had no exposure to iodinated CM. Forty-five (2.6%) of 1,729 patients had a previous history of ARs to CM and 164 (9.5%) had a history of allergy.

**Table 1:** Baseline characteristics.

Characteristic		
		n = 1,729
Age (y)	Median (IQR)	69 (62-74)
	Range	22-93
Male sex, n (%)		1,156 (66.9)
Absence of previous iodinated contrast media injections, n (%)		973 (56.3)
History of adverse reaction to iodinated contrast media, n (%)		45 (2.6)
History of allergy, n (%)		164 (9.5)
History of asthma, n (%)		23 (1.3)
Use of corticosteroid at entry, n (%)		28 (1.6)
Child–Pugh classification, n (%)		
A		1427 (82.5)
B or C		302 (17.5)
Renal function at entry		
eGFR (ml/min), Median (IQR)		73.6 (61.7-87.3)
Presence of renal impairment, n (%)		383 (22.2)

### Manifestations and severity of adverse reactions

A total of 23,684 iodinated CM examinations were performed in the 1,729 patients during the study period; the total number of ARs were recorded for 196 (0.83% on an injection basis and 11.3% on a patient basis) examinations.

The manifestations and severity of the recorded ARs are summarized in Table 2. Sixty-two patients developed hypersensitivity reactions. The most common manifestation of a hypersensitivity reaction was urticaria, which occurred locally in 25 patients and systemically in nine. There were two severe ARs: both were anaphylactoid shock. Seventy-one patients developed physiologic reactions. The most common symptoms were digestive manifestations, such as nausea or vomiting, which occurred in 35 patients. There were 58 other reactions and we could not know the manifestation of AR in 7 patients. Two patients developed both hypersensitivity and physiologic reactions (one had pruritus and dizziness, and another had nausea and pruritus). The number of CT examinations and TACE and the observed ARs are also provided separately in Table 2. Significantly more ARs were observed in TACE than during CT (0.78% vs. 1.60%, *P* = 0.0017). This difference was attributed to the number of physiologic reactions, in particular, vasovagal reflux (0.22% vs. 1.53%, *P* < 0.001).

**Table 2:** Manifestations and severities of overall adverse reactions (ARs) and ARs during contrast-enhanced computed tomography and transarterial chemoembolization.

			Overall		Enhanced CT		TACE		*P* value
			n = 23,684		n = 22,185		n = 1,499		
Patients with any immediate symptom, n (%)			196	(0.83)	172	(0.78)	24	(1.60)	0.0017
Hypersensitivity reaction, n (%)			62	(0.26)	61	(0.28)	1	(0.07)	0.19
Mild		Local urticaria, n (%)	25	(0.11)	25	(0.11)	0	(0.00)	0.40
		Pruritus, n(%)	8	(0.03)	8	(0.04)	0	(0.00)	1.00
		Scratchy throat, n (%)	7	(0.03)	7	(0.03)	0	(0.00)	1.00
Moderate		Systemic urticaria, n (%)	9	(0.04)	9	(0.04)	0	(0.00)	1.00
		Hypotension with tachycardia, n (%)	3	(0.01)	3	(0.01)	0	(0.00)	1.00
		Dyspnea/Bronchospasm, n (%)	8	(0.03)	8	(0.04)	0	(0.00)	1.00
Severe		Anaphylactoid shock with loss of consciousness, n (%)	1	(0.00)*	1	(0.00)*	0	(0.00)	1.00
		Anaphylactoid shock with convulsion, n (%)	1	(0.00)*	0	(0.00)	1	(0.07)	0.063
Physiologic reaction, n (%)			71	(0.30)	48	(0.22)	23	(1.53)	<0.001
Mild		Nausea/Vomiting, n (%)	35	(0.15)	29	(0.12)	6	(0.40)	0.021
		Dizziness, n (%)	5	(0.02)	5	(0.02)	0	(0.00)	1.00
		Flush, n (%)	4	(0.02)	4	(0.01)	0	(0.00)	1.00
		Sensation of warmth, n (%)	5	(0.02)	5	(0.01)	0	(0.00)	1.00
		Headache, n (%)	1	(0.00)*	1	(0.00)*	0	(0.00)	1.00
		Chest pain, n (%)	1	(0.00)*	1	(0.00)*	0	(0.00)	1.00
Moderate		Hypotension, n (%)	5	(0.02)	0	(0.00)	5	(0.33)	<0.001
		Hypertension, n (%)	1	(0.00)*	0	(0.00)	1	(0.07)	0.063
		Vasovagal reflux, n (%)	12	(0.05)	1	(0.00)*	11	(0.73)	<0.001
		Tachycardia, n (%)	1	(0.00)*	1	(0.00)*	0	(0.00)	1.00
		Severe vomiting, n (%)	1	(0.00)*	1	(0.00)*	0	(0.00)	1.00
Other, n (%)			58	(0.24)	58	(0.26)	0	(0.00)	0.051
	Sneezing, n (%)		39	(0.16)	39	(0.18)	0	(0.00)	0.18
	Cough, n (%)		19	(0.08)	19	(0.09)	0	(0.00)	0.63
Unknown, n (%)			7	(0.03)	7	(0.03)	0	(0.00)	1.00

Abbreviations: CT, computed tomography; TACE, transarterial chemoembolization.

* Less than 0.005%.

Note: Two patients had both hypersensitivity and physiologic reactions to a single exposure.

The difference in the adverse reaction rate was assessed by Fisher’s exact test.

**Figure 1:**
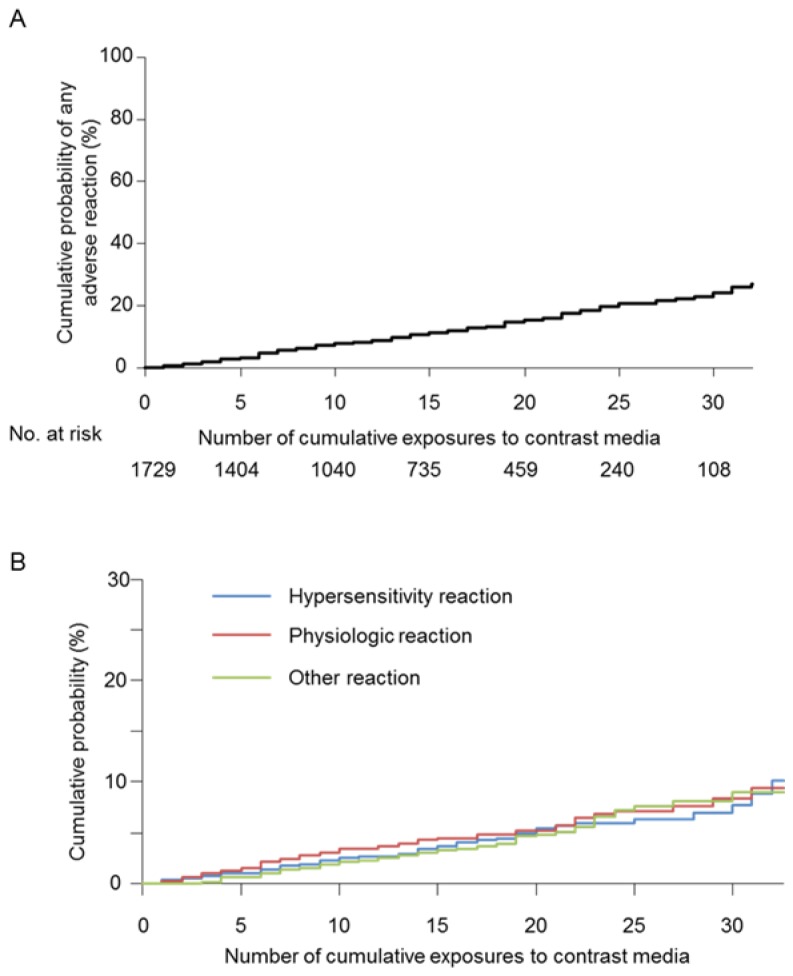
The cumulative probabilities of overall and each reactions to iodinated contrast media. Graphs illustrate (A) the cumulative probability of overall adverse reactions to iodinated contrast media and (B) the cumulative probabilities of hypersensitivity, physiologic and other adverse reactions to iodinated contrast media.

Additionally, we analyzed the cumulative probability of AR by drawing a cumulative probability curve over the number of cumulative exposures. The cumulative probabilities of overall immediate reaction at the 10th, 20th, and 30th contrast-enhanced CT examinations were 7.9%, 15.2%, and 24.1%, respectively (Figure 1A). Cumulative probabilities of a hypersensitivity reaction at the 10th, 20th, and 30th contrast-enhanced CT examinations were 2.5%, 5.4%, and 7.8%, respectively, whereas the cumulative probabilities of a physiologic reaction at the 10th, 20th, and 30th injections were 3.4%, 5.2%, and 8.4%, respectively. Cumulative probabilities of other reactions at the 10th, 20th, and 30th injections were 2.1%, 4.9%, and 9.0%, respectively (Figure 1B).

### Longitudinal changes in hazard of adverse reaction

**Figure 2:**
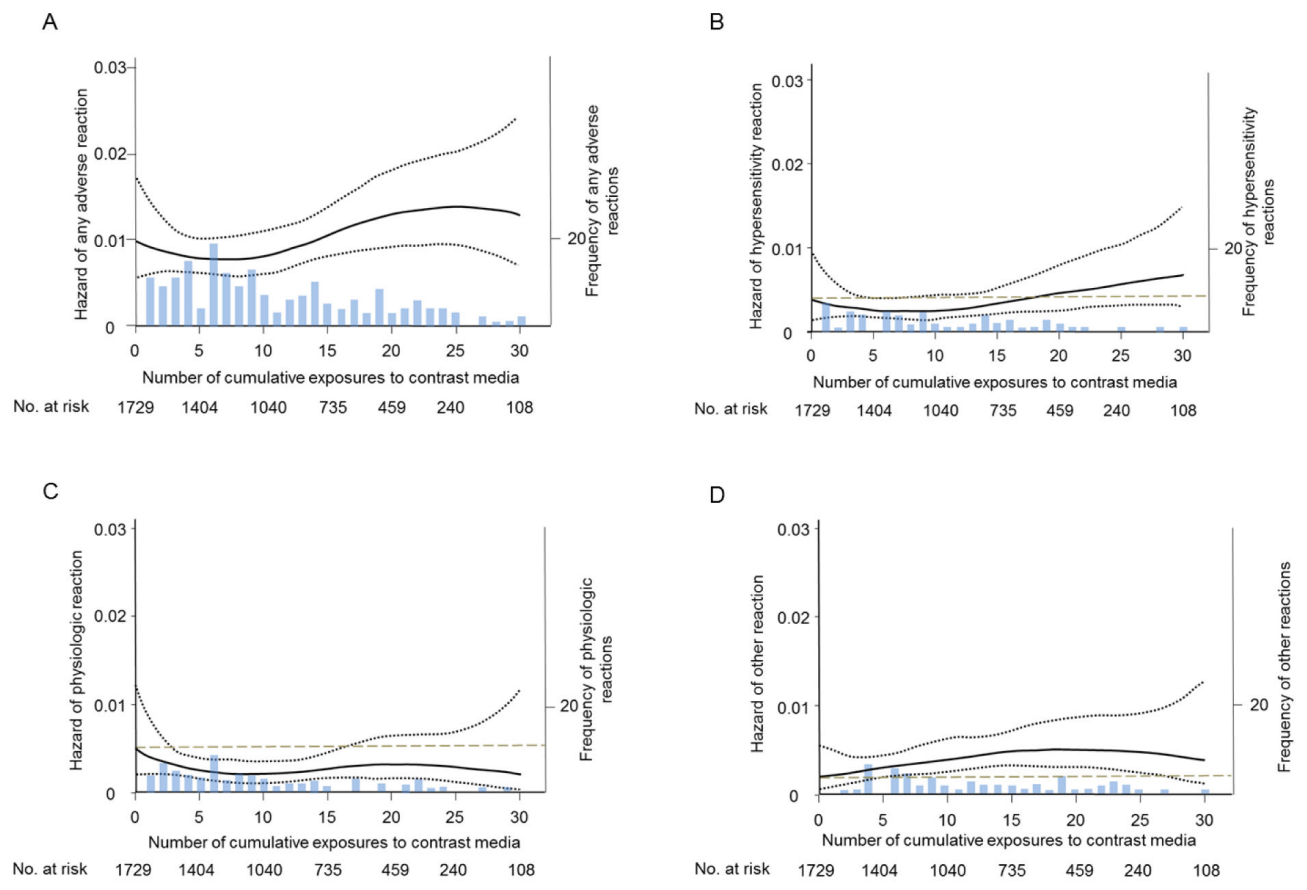
The smoothed plots of the estimated hazard of overall and each reactions to iodinated contrast media. The curves illustrate smoothed plot of the estimated hazard of (A) overall adverse reactions, (B) hypersensitivity, (C) physiologic and (D) other adverse reaction to iodinated contrast media in patients with no previous exposure to contrast media. The hazard function (solid line) and 95% confidence intervals (black dotted line) were estimated by a smoothing spline with three knots. Gray dotted lines show the hazard at the initial exposure.

We analyzed the hazard of ARs to CM in 973 exposure-naïve patients. The estimated hazard of overall AR gradually decreased until around the 10th exposure, and then increased thereafter (Figure 2A). The hazard function of hypersensitivity reaction demonstrated a V-shaped change with cumulative number of exposures (Figure 2B). The hazard function of physiologic reactions plateaued or decreased with increasing number of exposures to CM (Figure 2C). Risk of other reactions increased with cumulative number of exposures (Figure 2D).

### Risk factors for adverse reactions

**Figure 3:**
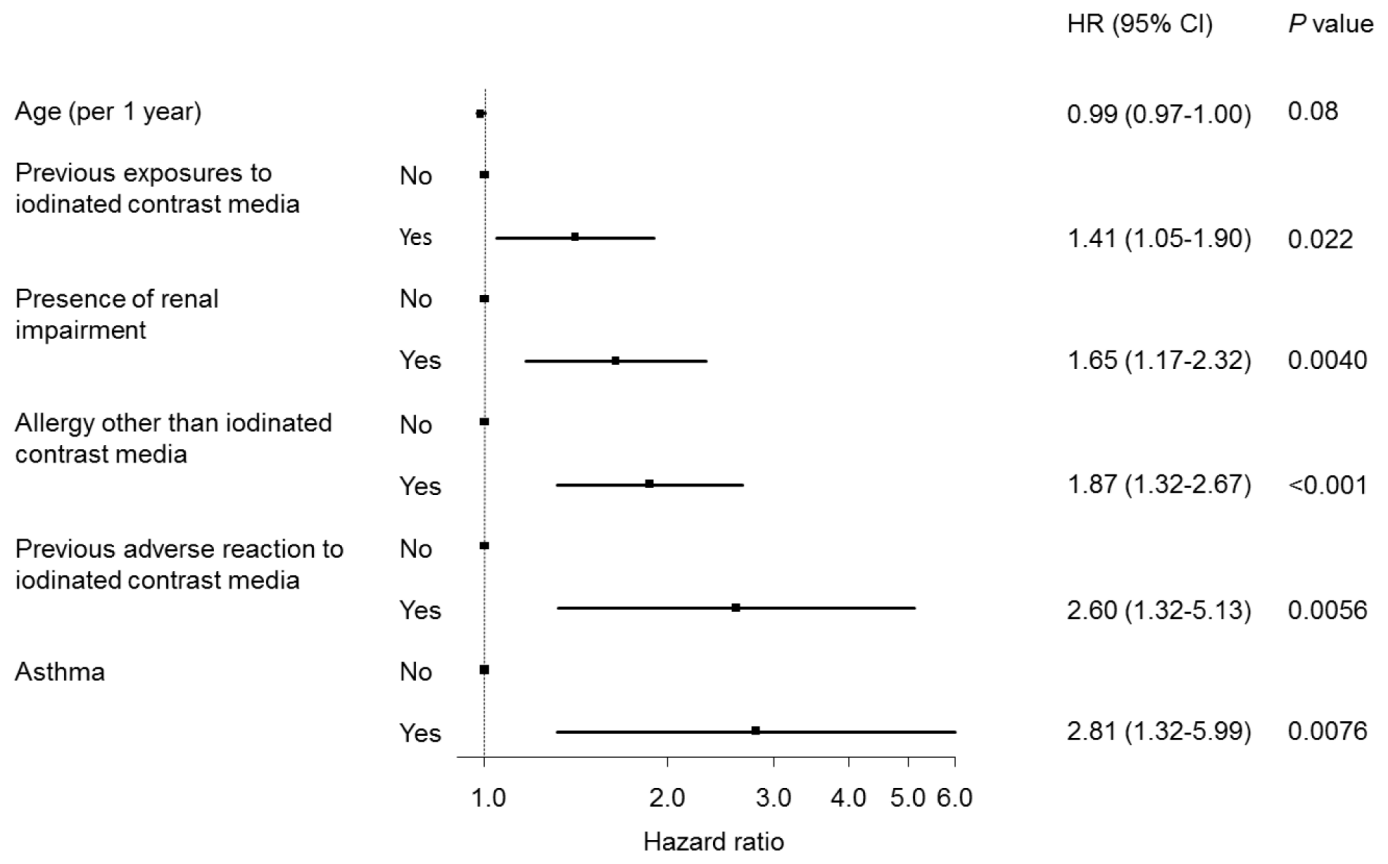
Forest plot of the hazard ratios for an initial adverse reaction during the study period.

In multivariate analysis with stepwise variable selection, the presence of previous iodinated CM exposure (HR: 1.41; 95% CI: 1.05, 1.90; P = 0.022), presence of renal impairment (HR: 1.65; 95% CI: 1.17, 2.32; P = 0.0040), previous AR to iodinated CM (HR: 2.60; 95% CI: 1.32, 5.13; P = 0.0056), past history of asthma (HR: 2.81; 95% CI: 1.32, 5.99; P = 0.0076), and past history of allergies other than iodinated contrast media (HR: 1.87; 95% CI: 1.32, 2.67; P <0.001) were found to be significant risk factors (Figure 3).

### Subsequent contrast media injections and adverse reactions to them

**Figure 4:**
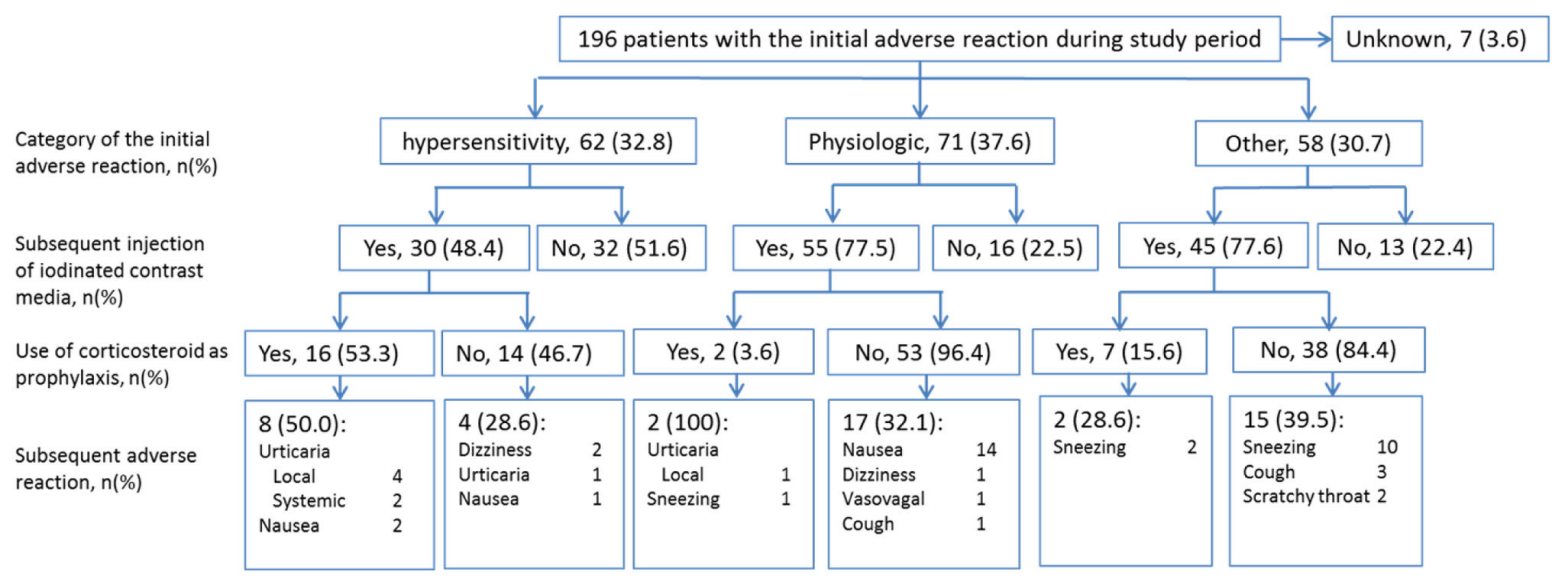
Subsequent contrast media injections and subsequent adverse reaction to iodinated contrast media.

Subsequent injections after the initial AR and ARs to subsequent injections are summarized in Figure 4. During the study period, initial ARs to iodinated CM occurred in 196 patients, 61 (31.1%) of whom did not receive further injections of iodinated CM. When patients with AR to iodinated CM were repeatedly re-injected, subsequent ARs occurred at high rates (36.9%). Re-injections of iodinated CM and ARs to them showed some trends. First, when the initial reaction was hypersensitivity, the patients were less likely to receive subsequent injections of iodinated CM (hypersensitivity vs. physiologic + other, 48.4% vs. 77.5%, respectively). When they did, corticosteroid was likely used as the prophylaxis when iodinated CM were re-injected (hypersensitivity vs. physiologic + other, 53.3% vs. 9.0%, respectively). Second, most recurrent ARs were classified into the same category as the initial reaction. The concordance rate for classification of the initial and subsequent reaction was 79.1%. Finally, only one case showed a subsequent AR that was more severe than the initial reaction. In this case, the patient had local urticaria as the initial reaction and systemic urticaria as the subsequent reaction. In contrast, two patients had subsequent reactions milder than the initial one. One patient had hypotension with tachycardia as the initial reaction and dizziness as the second. The other patient had systemic urticaria as the initial reaction and facial urticaria as the subsequent reaction with corticosteroid.

## Discussion

Although there are many reports of CM for CT, the current study is, to our knowledge, the first to report the cumulative effect of repeated iodinated CM injections on immediate ARs.

According to the hazard functions of AR analyzed in this study, the risk of ARs seemed to change biphasically (Figure 2A); the decreasing phase occurred during the first 10 injections, followed by an increasing phase. The decreasing phase may indicate that a portion of the patients were intrinsically sensitive to iodinated CM and had ARs at an earlier examination. In contrast, the increasing phase may reflect the cumulative effects of repeated CM injections.

By separate analyses for each AR category, we showed that the increasing phase consisted of hypersensitivity reaction and other reaction. Therefore, the risk of hypersensitivity reaction to iodinated contrast media accumulates with increasing number of exposures to CM. From the viewpoint of the cumulative effects of repeated exposures to CM, other reactions had similar properties to the hypersensitivity reactions. In this study, sneezing was classified as an ‛other’ reaction. However, given that paroxysmal sneezing is classified as a hypersensitivity reaction according to the ACR guidelines, a portion of the sneezing reactions may be caused by an allergic-like mechanism. Therefore, it was plausible that other reactions had similar properties to the hypersensitivity reactions.

In accordance with previous reports (8,18), the history of previous AR to CM, and the presence of asthma and other allergic diseases, were risk factors for ARs to CM. Patients with prior severe AR to CM or severe asthma were ineligible for CM injections and may have been excluded from this study cohort, resulting in a selection bias. Nevertheless, these variables were identified as risk factors for ARs to CM by use of a multivariate Cox proportional hazard regression model. This reinforced the strong associations between the ARs and these factors.

This present study also showed that the cumulative probability of ARs were significantly higher in patients with renal impairment than in patients with normal renal function (Figure 3). We could not explain the definite reason. However, we speculated that serum osmolality may increase at a relatively higher rate in patients with renal impairment than if a decrease in the eGFR level was due to hypovolemia, leading to strengthening osmotoxicity. Patients with renal impairment were expected to have fewer CM injections than patients with normal renal function because the renal function of such patients further deteriorated over a natural course, and these patients were contraindicated for CM injection. Thus, the incidence probability of ARs in patients with renal impairment may have been underestimated. Nonetheless, patients with renal impairment had significantly more frequent ARs than patients with normal renal function.

When patients with AR to iodinated CM were repeatedly re-injected with CM, subsequent ARs occurred at high rates (36.7%) (Figure 4). Previous reports showed that repeat CM reaction in premedicated patients, i.e., breakthrough reactions, were usually similar in severity to the initial reaction [29,30]. The results of this study are compatible with these previous reports.

This study has some limitations. First, the data set was obtained from a single institution and reviewed retrospectively, leading to an underestimation of the true incidence of ARs, due to the tendencies of retrospective investigations. Second, we admit that several patients may have received intravenous CM at other institutions, and developed an AR without our knowledge. Third, we did not evaluate other risk factors, such as the use of beta-adrenergic blockers [31,32]. Fourth, patients with the high risk factors such as severe asthma or other allergic diseases were possibly excluded from this study cohort. Fifth, this study has a low statistical power for very rare ARs like anaphylactoid shock. Sixth, we estimated the cumulative incidence and hazard of adverse reactions on the assumption that the risk of subsequent ARs is similar between TACE and contrast-enhanced CT, which is in fact controversial. The SCVIR study conducted by Bettmann et al. showed equal incidence of ARs after intra-arterial and intravenous administration of CM [33]. In contrast, others reported that intra-arterial administration of CM was accompanied by lower risk of ARs [34,35]. However, these reports estimated the risk of ARs to a single exposure only and did not take into account the impacts of repeated exposures. Therefore, we counted them together at the initial analysis and detailed differences in symptoms in Table 2.

In conclusion, the risk of immediate AR to iodinated CM increases with repeated exposures, and the incidence is strongly associated with renal function in patients with HCC. Not only renal toxicity but also AR to CM should be monitored carefully when iodinated CM are injected into HCC patients with mild renal impairment. However, even if patients with previous AR for iodinated CM were re-injected with iodinated CM, the patients with HCC had subsequent reactions at levels similar to the initial reaction.
