# Correction: Changes in Risk of Immediate Adverse Reactions to Iodinated Contrast Media by Repeated Administrations in Patients with Hepatocellular Carcinoma

**DOI:** 10.1371/journal.pone.0093340

**Published:** 2014-03-19

**Authors:** The PLOS ONE Staff

The name of the eighth author is spelled incorrectly. The correct name is: Koji Uchino.
